# New technique of local ischemic preconditioning induction without repetitive aortic cross-clamping in cardiac surgery

**DOI:** 10.1186/s13019-015-0206-0

**Published:** 2015-01-22

**Authors:** Dmitry I Kurapeev, Viktor O Kabanov, Vadim K Grebennik, Tatyana A Sheshurina, Vladimir V Dorofeykov, Michael M Galagudza, Eugene V Shlyakhto

**Affiliations:** 1 Institute of Experimental Medicine, Federal Almazov Medical Research Centre, Saint Petersburg, Russian Federation; 2 Institute of Heart and Vessels, Federal Almazov Medical Research Centre, Saint Petersburg, Russian Federation; 3 Department of Pathophysiology, First I.P. Pavlov Federal Medical University of St. Petersburg, Saint Petersburg, Russian Federation

**Keywords:** Local ischemic Preconditioning, Myocardial ischemia–reperfusion, Cardioplegia, Cardiopulmonary bypass, Troponin I, Cardiac index

## Abstract

**Background:**

Several studies have demonstrated that local ischemic preconditioning can reduce myocardial ischemia–reperfusion injury in cardiac surgery patients; however, preconditioning has not become a standard cardioprotective intervention, primarily because of the increased risk of atheroembolism during repetitive aortic cross-clamping. In the present study, we aimed to describe and validate a novel technique of preconditioning induction.

**Methods:**

Patients undergoing coronary artery bypass grafting (12 women and 78 men; mean age, 56 ± 11 years) were randomized into 3 groups: (1) Controls (n = 30), (2) Perfusion (n = 30), and (3) Preconditioning (n = 30). All patients were operated under cardiopulmonary bypass using normothermic blood cardioplegia. Preconditioning was induced by subjecting the hemodynamically unloaded heart to 2 cycles of 3 min of ischemia and 3 min of reperfusion with normokalemic blood prior to cardioplegia. In the Perfusion group, the heart perfusion remained unaffected for 12 min. Troponin I (TnI) levels were analyzed before surgery, and 12, 24, 48 h, and 7 days after surgery. The secondary endpoints included the cardiac index, plasma natriuretic peptide level, and postoperative use of inotropes.

**Results:**

Preconditioning resulted in a significant reduction in the TnI level on the 7th postoperative day only (0.10 ± 0.05 and 0.33 ± 0.88 ng/ml in Preconditioning and Perfusion groups, respectively, *P* < 0.05). In addition, cardiac index was significantly higher in the Preconditioning group than in the Control and Perfusion groups just after weaning from cardiopulmonary bypass. The number of patients requiring inotropic support with ≥ 2 agents after surgery was significantly lower in the Preconditioning and Perfusion group than in the Control group (*P* < 0.05). No complications of the procedure were recorded in the Preconditioning group.

**Conclusions:**

The preconditioning procedure described can be performed safely in cardiac surgery patients. The application of this technique of preconditioning was associated with certain benefits, including improved left ventricular function after weaning from cardiopulmonary bypass and a reduced need for inotropic support. However, the infarct-limiting effect of preconditioning in the early postoperative period was not evident. The procedure does not involve repetitive aortic cross-clamping, thus avoiding possible embolic complications.

## Background

Although the number of open heart surgeries performed each year has declined in the past decade, the proportion of very elderly patients with multiple comorbidities and/or multivessel coronary artery disease (CAD) undergoing major heart surgery is continuously increasing [1]. The peri- and postoperative mortality and morbidity has been reported to be increased in this patient population [2,3], which may be attributed—at least in part—to suboptimal myocardial protection due to a longer duration of aortic cross-clamping in advanced CAD [4], as well as the enhanced susceptibility of the aged and diabetic myocardium to ischemia–reperfusion injury (IRI) [5,6]. Therefore, the development and application of new cardioprotective approaches in cardiac surgery remains important.

Increased myocardial tolerance to IRI after a single episode or several brief episodes of ischemia–reperfusion is referred to as local ischemic preconditioning (IP) [7]. A large body of experimental evidence suggests that IP represents one of the most effective endogenous cardioprotective interventions developed thus far [8,9]. The predictability of ischemia onset and direct access to the heart are 2 major prerequisites for the successful clinical use of IP, both of which are fulfilled during open heart operations with cardiopulmonary bypass (CPB). At present, the cardioprotective effectiveness of IP in cardiac surgery patients has been demonstrated in several small clinical trials. In particular, IP has been shown to reduce the postoperative levels of cardiac troponins [10] and to improve left and right ventricular contractile function [11,12]. In addition, the beneficial effects of IP on certain clinical endpoints, such as the incidence of ventricular arrythmias [13], inotrope requirements [14], and intensive care unit (ICU) stay [15], have been clearly demonstrated. In all these studies, IP was induced after the initiation of CPB by 1 or 2 brief (1–5 min) cycles of aortic clamping followed by reperfusion prior to cardioplegic arrest. Although this protocol of IP induction seems to be safe in pediatric patients with congenital heart defects [16], repetitive aortic cross-clamping might be associated with an increased risk of atheroembolism in adult patients with diffuse atherosclerosis undergoing coronary artery bypass graft (CABG) surgery [17]. Thus, the development of alternative techniques of IP induction without repetitive (de)clamping of the ascending aorta is important.

In the present study, we describe a new technique of IP induction that does not require repeated aortic cross-clamping in patients undergoing cardiac surgery. In addition, we assessed the effects of the modified IP technique on serum cardiac troponin I (cTnI) and brain natriuretic peptide (BNP) levels, cardiac index (CI), left ventricular function, and clinical parameters in CABG patients.

## Methods

This single-center, double-blinded, randomized controlled study conforms to the principles outlined in the Declaration of Helsinki and UCL Good Clinical Practice Guidelines. The study design was approved by the ethics committees at Federal Almazov Medical Research Centre, St Petersburg, Russian Federation. Written informed consent was obtained from each patient before the operation.

### Inclusion/exclusion criteria

From March 2010 to October 2013, 90 patients (12 women and 78 men; mean age, 56 ± 11 [standard deviation] years) with 3-vessel CAD admitted for elective CABG surgery were enrolled in the prospective study. Patients with severe non-coronary cardiac disease, left ventricular ejection fraction < 50%, unstable angina, or history of myocardial infarction within 3 months prior to surgery were excluded. Patients with diabetes mellitus and those receiving inotropic agents prior to CPB were also excluded.

### Anesthesia and surgical technique

Premedication consisted of oral benzodiazepines and intramuscular morphine (0.15 mg/kg) given 1.5 h preoperatively. Before anesthesia, the right radial artery was cannulated and a Swan–Ganz pulmonary artery catheter was introduced through the right jugular vein. The patients were anesthetized by intravenous infusion of fentanyl and propofol. Pipecuronium bromide (0.15 mg/kg) was used for muscle relaxation. The activated clotting time was maintained at >450 s. CPB was established with a single 2-stage venous cannula and ascending aortic cannula. During CPB, the core body temperature was maintained at 36.0°С ± 0.5°С. The pump volume flow rate was 2.4–2.6 L/min/m^2^, and mean arterial pressure was maintained between 60 and 80 mm Hg. Intraoperative protection of the heart was performed using intermittent ante-retrograde warm blood cardioplegia. Antegrade cardioplegia was performed through the cannula positioned in the aortic root. Left ventricular venting was accomplished in all patients through the right superior pulmonary vein. The coronary sinus was cannulated with a self-inflating cannula for retrograde cardioplegia delivery. The ascending aorta was cross-clamped above the origin of the coronary arteries, followed by the ante-retrograde cardioplegia using a blood-based cardioplegia solution containing 20 mmol/L of potassium chloride. Oxygenated blood for cardioplegia was taken via the special outlet port of the oxygenator and delivered into the aortic cannula or coronary sinus using peristaltic pump. Before entering the vessels, the blood was supplemented with 10% solution of potassium chloride (KCl) via T-connector using the syringe pump. The volume flow rate of blood-based cardioplegia was kept at 200 ml/min during both induction of cardioplegia and maintenance of cardioplegic arrest (Table 1). The first episode of cardioplegia consisted of 2.5-min period of antegrade heart perfusion followed by 3.0-min period of retrograde perfusion. The beginning of antegrade delivery of blood was accompanied by bolus injection of 4 ml 10% KCl in the cardioplegia line followed by its infusion at a rate of 100 ml/h. During retrograde delivery of blood, the rate of KCl solution infusion was 10 ml/h. The blood cardioplegia solution containing 8 mmol/L of potassium chloride was infused for maintenance of cardiac arrest with an interval of approximately 15 min. The decrease in potassium concentration in the final cardioplegic solution was partially compensated by longer durations of subsequent (4^th^ and 5^th^) episodes of cardioplegia (Table 1). The rationale for decreasing potassium concentration was to avoid hyperkalemia-induced myocardial injury and accelerate recovery of myocardial contractility during reperfusion.

**Table 1:** **Protocol of intermittent ante-retrograde warm blood cardioplegia**

Episode of cardioplegia (No) and mode of infusion	Volume flow rate of blood delivery (ml/min)	Volume flow rate of delivery of 10% KCl solution (ml/h)	Duration of cardioplegia infusion (min)
1 - antegrade*	200	100 (preceded by bolus dose of 4 ml)	2.5
1 - retrograde	200	10	3.0
2 - retrograde	200	10 (preceded by bolus dose of 2 ml)	3.0
3 - retrograde	200	10	3.0
4 - retrograde	200	10	4.0
5 - retrograde	200	10	5.0

*first episode of cardioplegia consisted of 2.5-min antegrade infusion plus 3.0-min retrograde infusion.

The pressure in the coronary sinus was monitored throughout the procedure and was kept below 60 mm Hg. During antegrade cardioplegia, the pressure in the ascending aorta was monitored by the manometer and manual inspection of the aorta was performed to verify aortic valve closure.

Infusion of vasopressor (mesatone) was used in the patients with systemic vascular resistance of less than 800 dynes/s/cm^5^. Epinephrine, norepinephrine, and dopamine were used for inotropic support. Patients received inotropes either based on the observation of reduced cardiac contractility during and after weaning from CPB (by direct visual inspection of the right ventricle and/or transesophageal echocardiography) or after measuring a reduced CI (<2.0 L/min/m^2^), or both.

### Preconditioning protocol

All patients were randomized into one of the following 3 groups: (1) Control (n = 30); (2) Perfusion (n = 30); and (3) IP (n = 30). Randomization was performed in the surgery room; a sealed, nontransparent protocol envelope was opened for each patient and the patient was accordingly assigned to a group. The Control group included patients undergoing conventional CABG surgery. In the Perfusion group, a 12-min period of CPB was instituted before the application of the aortic cross-clamp in order to account for the effect of additional CPB time required for IP induction on the outcomes. In the IP group, preconditioning was elicited by cross-clamping the aorta and leaving the hemodynamically unloaded heart in the ischemic state for 3 min. During preconditioning ischemia, the left vent was run at 50 ml/min. A 3-min episode of ischemia was followed by 3 min of reperfusion with normokalemic blood delivered in the aortic root at a rate of 300 ml/min. The second episode of 3 min of ischemia was initiated by the cessation of re(perfusion) through the antegrade cardioplegia cannula. The full IP protocol included 2 cycles of 3 min of ischemia plus 3 min of reperfusion. The second 3-min episode of reperfusion was followed by the initiation of standard ante-retrograde blood cardioplegia.

### Measurements

The primary endpoint of the study was postoperative cTnI release. Blood samples for cTnI determination were obtained from each patient before the institution of CPB as well as 2, 6, 12, 24, and 48 h after surgery. Additional blood sampling was performed 7 days after surgery. BNP levels were determined preoperatively as well as on the 1st and 7th postoperative day. Serum levels of cTnI and BNP were measured using a chemiluminescent immunoassay (Architect i2000SR, Abbott Diagnostics, USA).

Left ventricular ejection fraction, left ventricular end-systolic volume, left ventricular end-diastolic volume, and left ventricular end-systolic and end-diastolic diameters were measured using echocardiography before surgery and on the 7th postoperative day. The cardiac index (CI) was calculated using the following formula: CI = cardiac output (l/min)/body surface area (m^2^). CI was calculated in all patients at 5 time points: (1) baseline (before induction of anesthesia); (2) prior to CPB; (3) after weaning from CPB; (4) 12 h after surgery; and (5) 24 h after surgery.

The duration of mechanical ventilation and length of stay in the ICU were recorded. The number of patients requiring inotropic support with ≥2 agents, mean number of inotropic drugs used, and mean total duration of inotrope use (h) were also recorded. The physicians in ICU and Echo lab were blinded to the group assignments. Postoperative morbidity and mortality on day 20 after the operation were recorded.

### Statistical analysis

Statistical analysis was performed using the SPSS 12.0 software package. All data in the text are expressed as mean ± standard deviation (SD). The sample size per group was determined using the following parameters: SD values calculated on the basis of previous studies, desired confidence level (95%), and acceptable difference in outcome between the groups (Statistics Calculator). The Kruskal-Wallis test was used to determine differences in the parameters analysed, followed by pairwise inter-group comparisons at each time point performed using non-parametric Mann–Whitney *U* tests. Fisher’s exact test was used to determine whether there were any differences between the groups in the number of patients requiring inotropic support with ≥2 agents. *P* values of ≤0.05 were considered significant.

## Results

### Perioperative characteristics of the patients

The preoperative data of the patients were similar between the 3 groups. There were no significant differences in age, sex, New York Heart Association class, previous myocardial infarction, and comorbidities such as peripheral vascular disease and chronic obstructive pulmonary disease among the groups. The CPB time, aortic cross-clamping time, total duration of cardioplegia infusion, number of distal anastomoses and duration of mechanical ventilation were also similar among the groups (Table 2).

**Table 2:** **erioperative characteristics of the patients**

Characteristic	Control (n = 30)	Perfusion (n = 30)	IP (n = 30)
Age (years)	55.4 ± 5.2	57.3 ± 5.6	56.6 ± 4.3
Gender (male/female)	5/25	3/27	4/26
Previous myocardial infarction	18 (60%)	20 (67%)	18 (60%)
NYHA class II	21 (70%)	22 (73%)	23 (77%)
NYHA class III	9 (30%)	8 (27%)	7 (23%)
COPD	11 (36%)	10 (33%)	9 (30%)
PAD	5 (17%)	6 (20%)	5 (17%)
CPB time (min)	86 ± 28	98 ± 29	89 ± 32
Aortic cross-clamping time (min)	48 ± 16	49 ± 19	52 ± 23
Total duration of cardioplegia (min)	9.8 ± 2.5	9.8 ± 3.2	9.9 ± 3.6
Number of distal anastomoses	3.2 ± 0.8	3.2 ± 0.5	3.4 ± 0.7
Graft material (LIMA/saphenous vein/radial artery) (%)	100/100/13.3	100/100/13.3	100/100/16.7
Duration of mechanical ventilation (h)	14 ± 6	14 ± 4	12 ± 3

Data are mean ± standard deviation. COPD – chronic obstructive pulmonary disease; CPB – cardiopulmonary bypass; IP – local ischemic preconditioning; LIMA – left interior mammary artery; NYHA – New York Heart Association; PAD – peripheral arterial disease.

### cTnI and BNP levels

According to the manufacturer’s recommendations, the 99th percentile reference cutoff for the cTnI level was 0.3 ng/mL. Prior to surgery, cTnI levels were well below the upper reference limit in all groups (Table 3). The maximum release of cTnI was observed between 6 and 24 h after surgery, followed by a progressive decrease in concentration. There were no differences in cTnI levels between the groups except for the cTnI level at the 7th postoperative day, which was significantly lower in the IP group than in the Perfusion group (*P* < 0.05, Table 3). The pre- and postoperative serum BNP levels are presented in Table 4. Although surgery resulted in significant elevation of BNP concentration, the BNP levels did not differ between the groups at any time point.

**Table 3:** **Pre- and postoperative serum levels of cardiac troponin I (ng/ml)**

Time point	Control (n = 30)	Perfusion (n = 30)	IP (n = 30)
Before surgery	0.004 ± 0.008	0.005 ± 0.011	0.010 ± 0.012
2 h after surgery	0.84 ± 0.79	1.61 ± 1.68	1.18 ± 0.77
6 h after surgery	1.48 ± 0.83	1.57 ± 0.78	1.65 ± 0.98
12 h after surgery	1.52 ± 0.62	1.57 ± 0.89	1.56 ± 0.61
24 h after surgery	1.67 ± 1.03	1.92 ± 2.45	1.17 ± 0.56*
48 h after surgery	0.97 ± 0.61	1.32 ± 1.83	0.75 ± 0.42
7 days after surgery	0.19 ± 0.23	0.33 ± 0.88	0.10 ± 0.05*

Data are mean ± standard deviation. IP – local ischemic preconditioning. *P < 0.05 versus Perfusion.

**Table 4:** **Pre- and postoperative serum levels of brain natriuretic peptide (ng/ml)**

Time point	Control (n = 30)	Perfusion (n = 30)	IP (n = 30)
Before surgery	41.6 ± 25.5	33.7 ± 25.1	43.8 ± 40.1
24 h after surgery	268.2 ± 129.2	310.2 ± 210.3	267.0 ± 200.3
7 days after surgery	147.5 ± 59.5	171.0 ± 98.2	160.0 ± 82.6

Data are mean ± standard deviation. IP – local ischemic preconditioning.

### Left ventricular function

The pre- and postoperative echocardiographic parameters of left ventricular function did not differ among the groups (Table 5). The preoperative values of CI also did not differ among groups (Table 6). CI tended to decrease after induction of anesthesia and increased after weaning from CPB. Immediately after weaning from CPB, the CI was significantly higher in the IP group compared with the Control and Perfusion groups (*P* < 0.01, Table 6). At 12 and 24 h after surgery, the CI was lower in the Perfusion group than in the Control group (*P* < 0.05). IP resulted in a significantly higher CI at 24 h after surgery compared with that in the Perfusion group (*P* < 0.05).

**Table 5:** **Pre- and postoperative echocardiographic parameters of left ventricular function**

Time point	Control (n = 30)	Perfusion (n = 30)	IP (n = 30)
Left ventricular ejection fraction, %			
Before surgery	63.7 ± 6.2	63.6 ± 6.3	61.6 ± 5.9
7 days after surgery	63.0 ± 6.6	61.9 ± 7.0	62.5 ± 6.7
Left ventricular end-systolic volume, mL			
Before surgery	50.4 ± 15.1	49.7 ± 15.1	53.3 ± 14.8
7 days after surgery	48.2 ± 11.9	50.8 ± 15.3	50.5 ± 14.2
Left ventricular end-diastolic volume, mL			
Before surgery	137.3 ± 21.9	134.6 ± 22.1	137.1 ± 18.7
7 days after surgery	130.1 ± 13.7	130.9 ± 22.5	134.0 ± 19.7
Left ventricular end-systolic diameter, mm			
Before surgery	35.4 ± 4.2	34.5 ± 4.5	34.2 ± 3.7
7 days after surgery	33.6 ± 3.3	34.8 ± 4.1	34.3 ± 3.6
Left ventricular end-diastolic diameter, mm			
Before surgery	53.0 ± 3.0	52.7 ± 3.7	53.2 ± 3.7
7 days after surgery	51.9 ± 2.2	52.4 ± 3.8	52.6 ± 3.2

Data are mean ± standard deviation. IP – local ischemic preconditioning.

**Table 6:** **Pre- and postoperative values of cardiac index (L/min/m** ^**2**^ **)**

Time point	Control (n = 30)	Perfusion (n = 30)	IP (n = 30)
Before anesthesia	2.7 ± 0.4	2.6 ± 0.4	2.7 ± 0.3
Prior to CPB	1.9 ± 0.2	2,0 ± 0.4	1.9 ± 0.2
After weaning from CPB	3.0 ± 0.2	2.9 ± 0.3	3.4 ± 0.3**^#^
12 h after surgery	3.1 ± 0.7	2.8 ± 0.4*	2.9 ± 0.6
24 h after surgery	3.0 ± 0.5	2.7 ± 0.3*	2.9 ± 0.3&

Data are mean ± standard deviation. CPB – cardiopulmonary bypass; IP – local ischemic preconditioning. *P < 0.05 versus Control; **P < 0.01 vs. Control; ^#^*P* < 0.01 vs. Perfusion; & – *P* < 0.05 vs. Perfusion.

### Postoperative course and inotrope requirement

None of the patients experienced perioperative myocardial infarction or stroke. No complications of the procedure were recorded in the IP group. There was 1 case of resternotomy in the Perfusion group because of persistent bleeding. Most of the patients were transferred from the ICU to the regular ward on the 1st postoperative day. There were 5, 7, and 2 patients who were transferred from the ICU on the 2nd day after surgery in the Control, Perfusion, and IP groups, respectively. One patient in the Control group and 1 patient in the Perfusion group were transferred from the ICU on the 3rd postoperative day. The reasons for prolonged postoperative stay in the ICU were heart failure, respiratory failure, and encephalopathy. The number of patients requiring inotropic support with ≥2 agents after surgery was significantly lower in the IP and Perfusion group than in the Control group (*P* < 0.05, Table 7). The mean number of inotropic drugs used per patient, as well as the mean total duration of inotrope use and inotrope doses, were similar in all the groups (Table 7).

**Table 7:** **Parameters of postoperative inotrope requirements**

Parameter	Control (n = 30)	Perfusion (n = 30)	IP (n = 30)
No of patients treated with ≥ 2 agents	8	5*	4*
Mean number of inotropic drugs	0.7 ± 0.8	0.5 ± 0.7	0.5 ± 0.7
Mean total duration of inotrope use (h)	8.8 ± 12.1	7.6 ± 13.4	6.6 ± 8.9
Mean dose of epinephrine (μg/kg/min)	0.014 ± 0.022	0.010 ± 0.019	0.008 ± 0.017
Mean dose of norepinephrine (μg/kg/min)	0.035 ± 0.025	0.028 ± 0.036	0.051 ± 0.045
Mean dose of dopamine (μg/kg/min)	0.75 ± 1.57	0.35 ± 1.14	0.89 ± 1.83

Data are mean ± standard deviation. CPB – cardiopulmonary bypass; IP – local ischemic preconditioning. *P < 0.05 versus Control.

## Discussion

Despite significant progress in the development of intraoperative myocardial protection techniques, including both optimization of cardioplegia regimens and implementation of new cardioplegia solutions [18,19], high-risk patients may develop severe myocardial IRI resulting in low cardiac output syndrome, left ventricular stunning, life-threatening ventricular arrhythmias, and perioperative myocardial infarction [20-22]. Considering the results of the numerous experimental studies, IP could be useful in reducing these manifestations of cardiac IRI. The first encouraging data on the effectiveness of IP in the settings of cardiac surgery were obtained by Yellon et al. [23] in 1993, who showed that 2 cycles of 3 min of global ischemia and 2 min of reperfusion prior to a 10-min episode of cross-clamp fibrillation without cardioplegia resulted in better preservation of tissue ATP compared with controls. Two subsequent studies by the same group have demonstrated that a similar IP protocol reduces postoperative cardiac troponin T (cTnT) levels [10,24]. An IP-mediated reduction in postoperative creatine kinase MB (CK-MB) levels has been found after antegrade cold crystalloid [25] and blood [14,26] cardioplegia. The infarct-limiting effect of IP was also verified by a significantly decreased cTnT and cTnI release in the patients undergoing open heart surgery with cardioplegia [27,28]. In addition, IP has been found to improve several important clinical parameters, including the incidence of arrhythmia [14,26,29], duration of artificial ventilation [28], and inotrope use [11,14,26]. Another group of endpoints for myocardial protection comprises left and right ventricular hemodynamic parameters. Improved left ventricular contractility during the postoperative period due to IP application has been found in several studies [11,25]. It has been also shown that IP can improve not only the left ventricular function, but also the right ventricular ejection fraction [13].

According to the results of systematic review of Heusch [30], 50% (8 out of 16) of IP studies in cardiac surgery demonstrated a significant reduction in infarct size, whereas the remaining studies reported either a neutral or even negative outcome. Taking into account the lack of significant infarct size limitation due to IP in the present study, it seems to be important to analyze the subset of IP studies failing to show a reduction in myocardial injury. A significant increase in the level of CK-MB was observed after the application of 2 cycles of 3 min of ischemia and 2 min of reperfusion prior to the onset of continuous retrograde warm cardioplegia [31]. The negative result of this study might be well attributed to the lack of test (prolonged) myocardial ischemia, whereas the increased level of CK-MB in the IP group reflects the sublethal myocardial injury caused by the preconditioning procedure itself. Kaukoranta et al. [32] noted a trend toward increased levels of CK-MB and cTnT after IP—an observation that could also be explained by the insufficient severity of test ischemia since this study utilized an almost uninterrupted coronary infusion of cardioplegia. The duration of myocardial ischemia was only 9% and 8% of the total cross-clamp time in IP and control groups, respectively. It should be noted, however, that IP failed to limit infarct size in several studies utilizing intermittent cold blood cardioplegia associated with an appreciable duration of test ischemia [33,34]. Nonetheless, these data led to the major conclusion that the limitation of significant infarct size through IP is more likely to be obtained in high-risk patients with the longest aortic cross-clamp times and severe left ventricular hypertrophy [35]. These patients are more likely to sustain significant perioperative myocardial injury during surgery, which might be potentially attenuated by protective maneuvers such as IP. Clinical data also indicate that IP may be especially useful for the preservation of right ventricular function because the right ventricle may be suboptimally protected during ante-retrograde cardioplegia [13].

The protocol for IP is crucial for effective myocardial protection. Experimental studies have indicated that the infarct-limiting effect of IP is generally proportional to the number of brief preconditioning stimuli, whereas the application of a single IP episode results in a marginal protective effect [36]. In this regard, the lack of IP effect on myocardial infarct size in some clinical trials could be, at least in part, attributed to the application of a single preconditioning episode instead of repeated episodes [11,32,37]. To our knowledge, none of the clinical studies on IP effects in cardiac surgery used an IP protocol consisting of >2 episodes of brief ischemia–reperfusion. The likely explanation for this fact is that repetitive aortic cross-clamping and subsequent declamping significantly increases the risk of atheroembolism. It is known that even the standard procedure of aortic cross-clamping in the patients with advanced atherosclerosis of the ascending aorta is associated with microembolism of the brain vessels, resulting in a varying degree of neurocognitive impairment [38,39]. Therefore, it is not surprising that the increased risk of atheroembolism due to repetitive aortic cross-clamping is one of the major factors hindering the routine application of IP in cardiac surgery [17]. Of note, the results of meta-analysis including 22 clinical trials on IP in cardiac surgery (933 patients), showed that IP significantly reduced the incidence of arrhythmias, postoperative inotrope use, and duration of ICU stay, which was corroborated by the results of the present study [40]. The authors speculated that the most rigorous evidence for the protective effect of IP could be obtained if one could demonstrate a twofold decrease in mortality, which, however, would require an inclusion of ≥3800 patients in each arm of the study. The introduction of IP protocols based on the no-touch-to-aorta techniques may facilitate this task.

A further protocol-related factor that plays an important role in the expression of IP-mediated protective phenotype is the ratio between the duration of ischemic and reperfusion phase of preconditioning cycle. Experimental data clearly demonstrate that IP is most effective when the duration of the reperfusion episode is equal to that of the ischemic episode or exceeds it. The indirect evidence supporting the validity of this notion was derived from the results of a recent clinical trial on IP that showed no infarct size limitation after the application of 2 cycles of 2 min of ischemia and 1 min of reperfusion [41].

Several studies have tested the hypothesis that CBP itself may elicit a preconditioning-like response, primarily through the development of a low-grade systemic inflammatory response [42]. Important data supporting this concept were obtained by Ghosh and Galinanes [37], who found a significant cardioprotective effect of IP only in patients undergoing off-pump CABG and not in those subjected to cardioplegia or intermittent cross-clamp fibrillation. Subsequently, IP has been shown to exert a significant protective effect in other studies performed on CABG patients operated off-pump [43,44]. However, it seems that the preconditioning-like effect of CPB may still permit additional protection afforded by IP. This idea is supported by the large body of evidence demonstrating IP-induced limitation of infarct size and amelioration of clinical parameters in the patients undergoing CBP [30,40].

Although BNP measurements are not routinely used in the studies on myocardial protection, the data start to accumulate that BNP measurement might be also relevant for patients undergoing cardiac surgery. It is well established that BNP levels are severely increased after heart transplantation (e.g., [45]) including the patients without severe hemodynamic perturbations or allograft rejection. Postoperative plasma BNP is independent predictor of cardiac dysfunction after cardiac surgery [46]. Therefore, we were interested to see whether BNP can be used as a secondary biochemical marker of myocardial protection. Although we noted significant elevation of BNP at 24 h postoperatively in all groups, there were no inter-group differences.

In conclusion, the present study describes a new technique of IP induction in cardiac surgery patients. This technique does not require repetitive aortic cross-clamping, which makes the preconditioning procedure safe for the patients with advanced atherosclerosis. The application of this technique of IP was associated with certain benefits, including improved left ventricular function after weaning from CPB and a reduced need for inotropic support. However, the infarct-limiting effect of IP in the early postoperative period was not evident—this requires additional refinement of the IP protocol as well as the identification of patient categories that may benefit from the application of IP.

## Conclusions

The IP procedure described can be performed safely in cardiac surgery patients. The application of this technique of IP was associated with certain benefits, including improved left ventricular function after weaning from CPB and a reduced need for inotropic support. However, the infarct-limiting effect of IP in the early postoperative period was not evident. The procedure does not involve repetitive aortic cross-clamping, thus avoiding possible embolic complications.

## Abbreviations

BNP: Brain natriuretic peptide
CI: Cardiac index
CPB: Cardiopulmonary bypass
CABG: Coronary artery bypass graft
CAD: Coronary artery disease
CK-MB: Creatine kinase MB
ICU: Intensive care unit
IRI: Ischemia–reperfusion injury
IP: Ischemic preconitioning
TnI: Troponin I
